# Multidimensional Study of the Attitude towards Euthanasia of Older Adults with Mixed Anxiety-Depressive Disorder

**DOI:** 10.3390/healthcare12111078

**Published:** 2024-05-24

**Authors:** Luís Fonseca, Luísa Castro, Guilhermina Rêgo, Rui Nunes

**Affiliations:** 1 Bioethics Department, Faculty of Medicine, University of Porto, 4200-319 Porto, Portugal; guilherminarego@med.up.pt (G.R.); ruinunes@med.up.pt (R.N.); 2 Psychogeriatrics Unity, Psychiatry Department, Senhora da Oliveira Hospital Guimarães, 4835-044 Guimarães, Portugal; 3 Department of Community Medicine, Information and Health Decisions Sciences, MEDICIS, Faculty of Medicine, University of Porto, 4200-450 Porto, Portugal; luisacastro@med.up.pt; 4 Centre for Health Technology and Services Research (CINTESIS@RISE), Faculty of Medicine, University of Porto, 4200-450 Porto, Portugal

**Keywords:** depression, anxiety, elderly, euthanasia

## Abstract

Introduction: This study aims to verify if older adults with mixed anxiety-depressive disorder are more prone to euthanasia and identify factors that interfere with their satisfaction with health and capacity for well-informed decisions. Material and Methods: The study applied a paper questionnaire composed of a sociodemographic section and a battery of scales (to assess depression, anxiety, cognitive performance, suicide risk, therapeutic adhesion, functionality, loneliness, attitude towards euthanasia, decision pattern, personality, empathy, and health status) in the Psychogeriatric Unity of Senhora da Oliveira Hospital in Portugal. The sample was collected by convenience to include patients and controls of the same age. Six months later, a reassessment was performed. Patients and controls were compared using descriptive statistics and a multiple-regression model. Results: A total of 114 patients and 25 controls were included. Eighty-one point six percent of patients had four or fewer years of schooling. Contrary to controls, they presented mild depressive and anxiety symptoms, loneliness feelings, worse cognitive performance, a more fragile personality, higher personal distress, and a poorer health state. No statistically significant differences were found between controls and patients regarding their attitudes towards euthanasia. Patients more favourable to euthanasia had higher empathic concern, conscientiousness, and fantasy, and lower personal distress. Discussion and Conclusion: When addressing euthanasia in these patients, it is crucial to ensure they are fully self-determinate and that all the necessary treatment and support are available. It may not be the case when the educational level is low and a mild disease persists, significantly affecting their well-being and cognitive performance.

## 1. Introduction

Demographic winter is a reality worldwide. By 2050, the number of people aged 60 and older will double, and the number of people aged 80 years or older is expected to triple [1]. In the European Union (E.U.), according to data from January 2022, older people (aged 65 or over) were around 21.1% of the total population, with Italy (23.8%), Portugal (23.7%), Finland (23.1%), Greece (22.7%), and Croatia (22.5%) being the most aged countries [2].

Between 2018 and 2080, according to a central projection scenario, the ageing rate in Portugal will almost double, from 159 to 300 older adults for every 100 young people [3].

The demographic turnover will have a tremendous impact on several areas of our society [4], including mental health, as it is known that advanced age represents a risk factor for depression and anxiety in midlife and older adults [5]. Thereby, it cannot be surprising that mental illness, particularly depressive and anxiety disorders, is currently among the top ten leading causes of global burden [6], as this is expected to increase. The burden of a disease is calculated using the disability-adjusted life year (DALY). In Portugal, Alzheimer’s and other dementias, depression, and anxiety disorders appear in the fifth, sixth, and ninth positions, respectively, of the ten top causes of DALY [7]. The estimated prevalence of anxiety among Portuguese older adults is 9.6%, and depression is 11.8% [8]. The most common mental health conditions for older adults are depression and anxiety [9]. Consequently, as ageing becomes more prominent, more older adults will suffer from these disorders.

In addition, the Portuguese elderly present specific frailties that will add to the problem, namely a low educational and literacy level [10] (some only know how to sign their name and many are not able to perform ‘simple’ digital tasks, like handling a smartphone or texting) and loneliness. Sixty-eight point five percent of people aged 65 or over have between 0 and 4 years of schooling [11], and the number of people living alone has been increasing, with the number of single-person families consisting of an older adult representing the majority of these families [12]. Low education and illiteracy have been correlated with anxiety and depressive symptoms [8] and a higher risk of dementia [13,14], mainly when years of education reflect cognitive capacity [15]. Also, loneliness has been linked with an increased risk of developing depression [16], anxiety [17], and dementia [18]. One study even associated a higher cortical amount of amyloid with loneliness in cognitively normal elderly individuals [19].

Several risk factors for anxiety and depression in older adults have been identified (e.g., personality traits, poor self-perceived health), augmenting the complexity of the demanded treatments [20]. Furthermore, there are also specific regional-related differences that increase the probability of suffering from depression and anxiety. Southern European countries (e.g., Portugal, Spain) have more socioeconomic inequality and more late-life depression than Northern European countries (e.g., Denmark, Sweden), and this relationship was not mitigated by more significant individual income [21].

Thus, considering the negative impact of the demographic winter on the elderly mental health, it is of the utmost importance to discuss and study in advance euthanasia (the act of deliberately ending patients’ lives at their explicit request to relieve suffering) in this population, particularly when a growing number of countries are legalising it [22,23,24]. In May 2023, the Portuguese parliament decriminalised euthanasia for people with a fatal disease and unbearable suffering [25].

Several studies found that numerous factors relate to attitudes towards euthanasia, such as religion [26], empathic skills [27,28], personality [29,30,31], disease type and severity [32,33], loneliness [34], and educational level, and other psycho- and socioeconomic variables [29]. Also, suicide risk and euthanasia have been approached, but no relation was found between the two phenomena [35]. However, all these findings have been complex to compare across studies because of the variety of sample characteristics and outcome measures [29,30]. Humans are complex, and several determinants affect their beliefs and decision-making [33,36]. As such, it is of the utmost importance to do exhaustive comparative studies involving older adults with psychiatric disorders that include objective assessments of several of those determinants, mimicking, as best as possible, the complexity of humans’ beliefs and decision-making processes when euthanasia is considered.

Herein, we present a multidimensional study of the attitude towards euthanasia of the Portuguese elderly with mixed depression and anxiety disorder, a commonly underdiagnosed disease that meets particular characteristics to assess the most frequent psychopathological domains that often coexist in older adults: depression and anxiety [37]. The aim is to verify if these patients are more prone to euthanasia and identify specific needs and weaknesses that may interfere with their satisfaction with health and capacity for well-informed decisions. If studied and discussed beforehand, tailored euthanasia legislation can be elaborated and targeted prevention and treatment strategies implemented to increase the well-being and decision-making capacity of older adults with mental health disorders.

## 2. Material and Methods

### 2.1. Inclusion and Exclusion Criteria

After the proper approval by the Ethics Committee of the SOH (ref. 70/2020), the sample was collected in the consultation of the PU between 7 May 2021, and 30 November 2022, to include older patients (aged ≥ 65) diagnosed with mixed anxiety-depression disorder (ICD-10), stable co-morbidities (if present), and medicated in accordance with the presented symptoms and international guidelines (13th Edition of the Maudsley Prescribing Guidelines in Psychiatry). Patients with depressive and anxiety symptoms secondary to non-psychiatric illness, chronic pain, and dementia were omitted. Also, mentally healthy controls of the same age with stable co-morbidities (if present) and without any psychopharmacological medication were collected by convenience from the consultation of the PU (where healthy companions of the patients were asked to participate voluntarily) and the community. Six months later, a reassessment using the same instruments was performed. During this time, patients continued to be followed as usual in the PU consultation, which is based on a psychopharmacological and psychoeducational approach. Patients and controls were compared using descriptive statistics and a multiple-regression model.

### 2.2. Instruments

This study involved applying a paper questionnaire both in the community and in the Psychogeriatric Unit (PU) of the Psychiatry Department (PD) of Senhora da Oliveira Hospital (SOH) in Portugal. The questionnaire comprised a sociodemographic section and a battery of scales validated for the Portuguese population. The participants always filled out the questionnaire with a researcher available. If any doubts occurred, they were promptly clarified.

The purpose of the use of the several following instruments was to analyse which of the factors or combinations of them most influence the attitudes towards euthanasia of the participants, given the various determinants that operate in humans’ personal beliefs and decision-making processes, as stated previously.

The hospital anxiety and depression scale (HADS) comprises seven questions for anxiety and seven questions for depression, and cut-off scores are available for quantification (8–10: mild symptoms; 11–14: moderate symptoms; 15–21: severe symptoms). The instrument [38] was validated for the Portuguese population (α: anxiety = 0.76, depression = 0.81) [39] and revealed a Cronbach’s alpha in our sample of 0.889 for anxiety and 0.847 for depression.

The UCLA loneliness scale (UCLALs) is a 16-item scale designed to measure one’s subjective feelings of loneliness. Participants rate each item as either “I often feel this way”, “I sometimes feel this way”, “I rarely feel this way”, or “I never feel this way”. Scores > 32 indicate feelings of loneliness. UCLALs [40] was validated for the Portuguese population (α = 0.905) [41]. Our sample presents a Cronbach’s alpha of 0.953.

Treatment adherence was assessed using the Measure Treatment Adherence (MTA) scale. This scale is an instrument composed of seven items that assess an individual’s behaviour about the everyday use of medicines. The answers are obtained by an ordinal six-point scale ranging from ‘always’ (1 point) to ‘never’ (6 points). The values obtained from the responses to the seven items are added and divided by the number of items. Higher values mean higher levels of adherence. The scale is validated for the Portuguese population (α = 0.74) [42], and the Cronbach’s alpha in our sample was 0.831.

The Barthel index (B.I.) is an ordinal scale that measures functional independence in personal care and mobility [43]. The 10-item version is the most used. The scoring method considers whether the person receives help while doing each task. The scores for each of the items (0, 5), (0, 5, 10), or (0, 5, 10, 15), depending on the item] are summed to create a total score, with higher scores indicating higher levels of independence. It is validated for the Portuguese population (α = 0.622) [44]. The Cronbach’s Alpha in our sample was 0.622.

The Yara’s attitude towards euthanasia scale (YATEs) was validated with eight samples and applied in research studies with highly satisfactory results [45,46,47]. It assesses the overall tendency of a specific group regarding euthanasia and allows comparisons between groups. Scores range from 0 to 104. Higher scores indicate a more favourable attitude towards euthanasia. The sample median divides those with a more favourable attitude from those with a less favourable attitude. The Cronbach’s Alpha of the cross-cultural adaptation and validation for European-Portuguese was 0.934 [48] (α = 0.983 in our sample).

Wasserman’s (2005) attitude towards euthanasia scale (WATEs) is a 10-item scale that measures attitudes towards euthanasia, considering different dimensions: severe pain, the impossibility of recovery, the patient’s request, the physician’s authority, active euthanasia, and passive euthanasia. The subjects answer each one using the Likert scale response categories of (1) strongly disagree, (2) disagree, (3) undecided, (4) agree, and (5) strongly agree. The scale’s internal consistency in the original study was measured by a Cronbach’s alpha of 0.87 [49]. The internal consistency of the Portuguese version was good (Cronbach’s alpha = 0.90) [50]. Cronbach’s alpha was also analysed for two dimensions, “Decision/Will of the Patient” and “Decision/Evaluation of the Physician”, revealing a high internal consistency with values of 0.94 and 0.85, respectively [50]. In our sample, wATEs presented high internal consistency for both the total scale (α = 0.98) and its two dimensions (“Patient’s Decision/Will”: α = 0.97, and “Doctor’s Decision/Evaluation”: α = 0.98).

The Melbourne Decision-Making Questionnaire (MDMQ) was designed to assess how individuals approach decision situations [51]. It includes five subscales to which the respondent checks “True for me” (score 2), “Sometimes true” (score 1) or “Not true for me” (score 0). Each scale ranges from zero to 10 (procrastination and hypervigilance) or 12 (vigilance, self-esteem and buck-passing). It is validated for the Portuguese population [α (self-esteem) = 0.76; α (vigilance) = 0.747, α (buck passing) = 0.859, α (hypervigilance) = 0.782, α (procrastination) = 0.793] [52]. In our sample, Cronbach’s alpha was 0.838 for self-esteem, 0.911 for vigilance, 0.936 for buck passing, 0.833 for procrastination, and 0.812 for hypervigilance.

The NEO Five-Factor Inventory (NEO-FFI) concisely measures five personality factors (neuroticism, extraversion, openness, agreeableness, and conscientiousness), with 12 items for each factor [53]. Each of the items is measured on a Likert-based scale ranging from 0 (“Strongly Disagree”) to 4 (“Strongly Agree”). Higher scores in each domain indicate a higher impact of that particular personality trait. The Portuguese version of the NEO-FFI revealed good reliability with Cronbach’s alpha values (conscientiousness = 0.81, neuroticism = 0.81, extraversion = 0.75, agreeableness = 0.72, and openness = 0.71) related to the ones reported for the original NEO-FFI in the USA [54]. In our sample, the Portuguese NEO-FFI also presented high internal consistency in four of its five dimensions: conscientiousness = 0.848, neuroticism = 0.934, extraversion = 0.859, agreeableness = 0.618, and openness = 0.862.

The interpersonal reactivity index (IRI) is based on a multidimensional view of empathy and comprises four sub-scales: perspective-taking, empathic concern, personal discomfort, and fantasy [55]. For each statement/item of the IRI, a person is asked to indicate to what extent that statement applies to themselves, using a 5-level scale (between “Does not describe me well” and “Describes me very well” using the numbers 0 and 4, respectively, and 1, 2, and 3 for intermediate evaluations). The quotation is made by adding these values by sub-scale and averaging them. Higher scores indicate a higher capacity, use, or intensity of the respective component of empathy. The Cronbach’s alpha of the IRI scale in the Portuguese version (perspective taking = 0.74; empathic concern = 0.77; personal discomfort = 0.81; fantasy = 0.83) was moderate and similar to those found with the other versions of the IRI [56]. In our sample, the IRI presented the following Cronbach’s alpha: perspective taking = 0.936; empathic concern = 0.838; personal discomfort = 0.919; fantasy = 0.870.

The Short Form 36 Health Survey Version 2 (SF36v2) is a patient-reported outcome assessment designed to measure patients’ quality of life, functional health, and well-being across various conditions. It covers eight health domains: physical function, physical role, pain, general health, vitality, social function, emotional role, and mental health [57]. It was validated for the Portuguese population [58,59] presenting the following Cronbach’s’ alpha: α (physical function) = 0.8731; α (physical role) = 0.7511; α (pain) = 0.8441; α (general health) = 0.8745; α (vitality) = 0.8264; α (social function) = 0.6031; α (emotional role) = 0.7104; α (mental health) = 0.6446. In our sample, Cronbach’s alpha was: α (physical function) = 0.920; α (physical role) = 0.964; α (pain) = 0.833; α (general health) = 0.821; α (vitality) = 0.859; α (social function) = 0.844; α (emotional role) = 0.945; α (mental health) = 0.937.

The tool for assessment of suicide risk (TASR) has been designed to be used by clinicians to document a summary of their assessment of a patient who may be suicidal. The TASR is divided into three sections: individual profiles, symptom profiles, and interview profiles. The TASR is a ‘bedside’ tool that helps the clinician determine the ‘burden of risk’ for suicide [60]. Points are used to provide the clinician with a section weighing suicide risk. Section 1 is assigned a weighing of one point for each item; Section 2, two points; and Section 3, three points. The greater the number of points, the greater the level of suicide risk. The tool’s developers provided no psychometric properties or indication of its validity in assessing suicide risk. Nevertheless, we decided to use it here to have a concrete and brief measure of the suicide risk and to verify if it relates to a specific attitude towards euthanasia.

The mini-mental state [61,62] and clock-drawing tests [63,64] are two brief tests that assess an individual’s cognitive performance and are closely related to one’s education level [62,65]. In our patient’s sample, the correlations between schooling/mini-mental state (MMS) and schooling/clock-drawing test (CDT) were significant and positive [schooling/MMS: r(112) = 0.48 (*p* < 0.001); schooling/CDT: r(112) = 0.56 (*p* < 0.001)], showing that even slight differences in the years of education can have a significant impact on one’s cognitive abilities. In the MMS, the considered Portuguese normative values were: possible cognitive decline if MMS ≤ 22 for subjects with from 1 to 11 years of education, ≤27 for those with 11 years of education, and ≤15 for the illiterate. The CDT used a 10-point quantitative system encompassing three major clock components. The selected time setting was “11:10”, as recommended by several authors [66,67]. Scores ≤ 6 were considered abnormal.

## 3. Statistical Analysis

Analyses were performed using the statistical software package SPSS Statistics (v. 28.0; SPSS^®^ Inc., Chicago, IL, USA) and Jamovi 1.1.9.0 (datalab. CC, Sydney, Australia). The categorical variables were described by absolute and relative frequencies, *n* (%). The normality of quantitative variables was evaluated by visual inspection of the respective histograms and by the Kolmogorov–Smirnov test. The quantitative variables presented deviations from the normal distribution and were described by median and interquartile interval, Med [1stQ, 3rdQ]. The Chi-Square test was used to compare categorical variables between cases and controls, the Fisher–Freeman–Halton test was employed for categorical variables with more than 2 categories and small expected counts, and the Mann–Whitney U test was used to compare non-normally distributed quantitative or ordinal variables between the two groups. Correlations were calculated using the Spearman correlation coefficient, r. Values of *p* ≤ 0.05 were considered statistically significant.

A multiple regression analysis was performed in the patient’s sample to verify which variables were associated with the attitude towards euthanasia (outcome). First, all the relevant variables were tested in simple linear regression models for the outcome. Then, all variables significant at the *p* < 0.2 level in the simple models were included in an initial multiple model. The final model was obtained by successively eliminating the independent variables with the highest *p*-value until only variables significant at the 0.05 level remained. The results of linear regressions are presented with non-standardised coefficient values (B), 95% confidence intervals (95% CI), and *p*-values. Multiple models were evaluated using the F statistics, *p*-values, and coefficients of determination (R2). All final models complied with the adequate assumptions: normally distributed residuals (assessed by the Kolmogorov–Smirnov test), no multicollinearity (variance inflation factors < 2), and homoscedasticity (absence of patterns in the scatter plot of residuals versus unstandardized predicted values, with a consistent spread along the fitted values).

## 4. Results

### 4.1. Descriptive Analysis

One hundred and fourteen (114) patients and 25 controls participated in the study at baseline, and ninety patients and seventeen controls six months later. Dropouts were voluntary and for no other reason than not wanting to participate further.

At baseline, patients (P) and controls (C) were of the same age (P-median = 72; C-median = 7), and most were married (P-60.5%; C-84%), Catholic (P-97.4%; C-92%), retired (P-98.2%; C-100%), with a median household of two (P-44.7%; C-68%), and controls had more years of schooling (P-median = 4; C-median = 6). Seventy-six percent of patients were female, and 56% of controls were male (Table 1). Psychometric assessment (Table 2) showed that both patients and controls had good therapeutic adherence (MAT: patients-med = 5.86; controls-med = 6; *p* = 1) and were independent for daily life activities (BI: patients-—med = 100; controls-med = 100; *p* = 1). In addition, several statistically significant differences between controls and patients were found. Patients revealed mild depressive and anxiety symptoms (HADS—*p* < 0.001), loneliness feelings (UCLALs—*p* = 0.001), worse cognitive performance (MMS/CDT—*p* < 0.001), a more fragile and dysfunctional personality [more neurotic (*p* < 0.001) and less extroverted (*p* < 0.001), open (*p* < 0.008) and conscientious (*p* = 0.017) on NEO-FFI], higher personal distress (IRI personal distress = *p* < 0.001) and worse health state [(worse physical functioning (*p* < 0.001), physical role (*p* < 0.001), general health (*p* < 0.001), vitality (*p* < 0.001), social function (*p* < 0.001), emotional role (*p* < 0.001) and mental health (*p* < 0.001) on SF36-v2]. Patients’ reassessment six months later (Table 3) revealed an improvement in depressive and anxiety symptoms (HADS—*p* < 0.001), better cognitive performance (MMS/CDT—*p* < 0.001), fewer loneliness feelings (UCLALs—*p* < 0.001), and a better physical role (*p* = 0.010), social function (*p* = 0.007), emotional role (*p* = 0.001), and mental health (*p* < 0.001). However, the differences between controls and patients remained the same, except for conscientiousness (Table 2).

No differences were found between controls and patients regarding attitudes towards euthanasia, as measured by euthanasia YATEs and WATEs.

Patients tendentially more favourable to euthanasia presented higher perspective-taking capacity, empathic concern, fantasy, openness, agreeableness, conscientiousness, and vigilance, and lower personal distress, buck-passing, procrastination, and hypervigilance (Table 4). Controls more favourable to euthanasia presented higher perspective-taking capacity, empathic concern, and lower procrastination (Table 4).

### 4.2. Multiple Regression Analysis of the Patient’s Sample

All the variables with *p* < 0.2 in the simple model (Table 4) were included in the initial multiple regression model (Table 5).

Since some independent variables were highly correlated [corr (self-esteem; vigilance) = 0.803, corr (self-esteem; buck-passing) = −0.754, corr (procrastination; buck-passing) = 0.757, corr (buck-passing; hypervigilance) = 0.781, and corr (procrastination; hypervigilance) = 0.824], three were eliminated (self-esteem, procrastination, and hypervigilance). Thus, only vigilance and buck-passing were included in the multiple model.

The multiple model was obtained progressively, eliminating the non-significant (*p* < 0.05) variables. The only significant variable in the final model was perspective-taking, which alone explained around 65.7% of the variability of YATEs. For each point of the sub-scale perspective-taking, YATEs increased an average of twenty-six points. This happened because perspective-taking was highly correlated with the outcome (YATEs, r = 83%). A decision was then made to exclude this variable from the final model.

In the end, the significant associations with a favourable attitude towards euthanasia in the patients’ sample were higher conscientiousness, empathic concern, and fantasy, and lower personal distress (Table 5).

## 5. Discussion

Portugal recently legalised euthanasia for competent (as determined by a judge) people with a fatal disease and unbearable suffering [25]. It is only matter of time before this subject will be addressed for psychiatric disorders. Therefore, it is of the utmost importance to discuss and understand several issues surrounding mental illnesses among the most vulnerable segments of the Portuguese population, like older adults.

Euthanasia of psychiatric patients is not only about whether they are legally competent and self-determined to make such a request. Many are undoubtedly able to do so [68,69]. In our study, patients were shown to be independent in their daily life activities [B.I. mean, controls/patients (baseline and six months later) = 100; *p* = 1]. However, they had worse cognitive performance, more neuroticism, and less extraversion and openness at baseline and six months later (see MMS, CDT, and NEO-FFI in Table 2). In addition, people lose much of their autonomy when they grow old and fragile, becoming increasingly inclined or forced to leave decisions to others [70]. Decision-making capacity is linked to several individual characteristics, such as personality [71,72] and individual cognitive abilities [73], and mild cognitive impairment is associated with poorer decision-making [74]. Thus, it is doubtful whether, under certain conditions, older adults retain their total decision-making capacity (the ability to make a medical decision within a given situation) for free and informed consent in health. Several Portuguese elderly who have 4 years of schooling or less (in our study, 81.6% of patients and 44% of controls had 4 or fewer years of education) have minimal reading or writing skills, with some only knowing how to sign their name and many not being able to perform ‘simple’ digital tasks (e.g., handling a smartphone or texting). Therefore, there are cognitive limitations that may not be only the result of a mental illness but also of a poor socio-cultural and academic background [75]. This adds to the problem of the negative influence of depressive and anxiety symptoms on cognitive performance.

As shown in Table 2, the presence of mild mental illness (HADS scores) is sufficient to determine feelings of loneliness (despite most patients—76.3%—living in a household of two or more people), lower self-esteem, worse cognitive performance, personal distress and health satisfaction, and a higher suicide risk. Now, in countries where euthanasia is available for psychiatric patients claiming unbearable mental suffering, the legalisation is based on the assumption that there is no clinical or legal argument to consider physical suffering worse than mental suffering [76,77]. Hence, if intolerable mental anguish is advocated, the proper and legally established assessment for euthanasia can be initiated. However, two significant problems arise here. First, the quantification of “unbearable” mental suffering poses more incredible difficulty in comparison to “unbearable” physical suffering [78,79,80], leaving more space for subjectivity in the assessment and potentiating some arbitrariness regarding which cases are allowed or not. Imagine that a patient from our study, suffering from mild depression and anxiety, claimed unbearable suffering. Would euthanasia be admissible? Despite psychiatrists being well-trained doctors, there is a heterogeneity in mental health assessment [81] that can lead two psychiatrists to classify the severity of the same patient’s disorder differently. Secondly, in many cases, patients may not be offered or subjected to all the necessary treatments [82]. In mental health, therapies are holistic and go far beyond biological treatments [83,84]. Nevertheless, in daily clinical practice, and despite the recommendations of several official guidelines, the failure to respond to biological therapies is too often the only criterion to consider a psychiatric disorder refractory to treatment [85]. This is particularly important in older people, where psychosocial issues and ageing-related problems arise, increasing the need for non-pharmacological approaches [69,86]. In our study, six months after the first evaluation, upon psychiatric follow-up, there was an improvement in depressive and anxiety symptoms, leading to better cognitive performance, social function, and mental health and fewer feelings of loneliness (Table 3). However, this follow-up, with a medical-centred approach (psychopharmacology and brief counselling), was insufficient for a full recovery as the differences between patients and controls remained the same (Table 2).

Unlike other medical disciplines, where it is easier to establish analytical criteria for evaluation, diagnosis, and intervention, psychiatry is a grey area. That is, diseases (for which there are no biological markers), from a longitudinal perspective, are often dynamic in their nature and intensity [87]. Furthermore, there is significant individual variability about diseases’ aetiology and perpetuating factors, and the needs of each patient include complex and tailored bio-psycho-social interventions. In older adults, a clinically and socially idiosyncratic age group, physiological weaknesses and social losses accumulate, and the chronological proximity to death becomes progressively more self-aware. In addition, can it be said that patients who refuse evidence-based treatments (56% of Dutch patients who received physician-assisted death due to psychiatric suffering did refuse some therapy) suffer irremediably [88]? Older adults are particularly prone to the tiredness of life argument for requesting euthanasia without sufficient medical grounds for their suffering to be legally granted [89]. However, data show that the willingness to die without severe disease is often ambiguous and does not necessarily represent a genuine wish to die [90]. As the experience of the countries where euthanasia is available for people with mental illness shows us, psychiatric patients are increasingly seeking access to euthanasia [91]. Curiously, several patients do not complete the process, indicating that the formal request for medically assisted death is a way of getting attention and help [92].

Moreover, the stigma that hangs over mental illness can alienate patients, particularly older adults, depriving them of adequate support. This is congruent with our data, where no significant difference was found between patients and controls regarding their attitude towards euthanasia (Table 2). Despite the higher suicide risk, the disease does not determine a specific attitude towards euthanasia in these elderly patients, even when time passes and mild symptoms prevail. However, this persistent symptomatology, along with loneliness feelings and health dissatisfaction [as opposed to controls (Table 2)], may lead patients who may not have had access to the necessary holistic treatment to ask for euthanasia.

Finally, despite depressive and anxiety symptoms and loneliness feelings not appearing to determine a specific tendency towards euthanasia (Table 2), several factors are more related to a favourable attitude. Patients with higher perspective-taking, empathic concern, fantasy, openness, agreeableness, consciousness, self-esteem, and vigilance and lower personal distress, buck-passing, procrastination, and hypervigilance tend to be more favourable to euthanasia (Table 4). In controls, only higher perspective-taking, empathic concern, and lower procrastination correlated significantly with YATEs (Table 4). This is consistent with some studies where some of these variables, more or less consistently, have been associated with the same tendency (e.g., empathy [27,28] and personality traits [29,30]). However, none of these studies either analysed attitudes towards euthanasia in elderly patients with mixed anxiety-depression disorder nor did they objectively use several psychometric instruments, trying to mimic humans’ complexity. Because human beings are multidimensional and several traits, health conditions, and social factors influence their thoughts, opinions, and actions, a regression model was used in our study, and higher conscientiousness, empathic concern, and fantasy, and lower personal distress were identified as the variables that better explained a favourable patient attitude towards euthanasia. This might mean that patients with persistent depressive and anxiety symptoms, feelings of loneliness, a precarious health status, and a higher suicide risk as compared to controls when endowed with higher conscientiousness (which is related to enhanced cognitive abilities [93]), empathic concern and fantasy (which measure individuals’ tendency to imagine themselves in fictional situations and is associated with empathic accuracy [94]), and lower distress are more aware of their precarious situation and more inclined to consider euthanasia as a viable way out. Stimulating older people’s literacy and cognitive abilities is crucial, augmenting their autonomy and informed decision-making capacity. However, one must remember that it is crucial to put all the necessary support (clinical, psychosocial, and economic) at their disposal to correspond to those higher demands. Otherwise, a sense of dissatisfaction could be nurtured, and euthanasia could be perceived as a fast, unique, and painless solution.

Some limitations should be noticed in our study. First, large samples are advised to reduce measurement errors and produce generalisable results for the same population. Second, the participants’ low literacy levels might have difficulty accurately interpreting the statements and questions of the psychometric instruments used, producing results bias. Third, longitudinal assessment should be interpreted cautiously as the number of participants, particularly controls, decreased, diminishing the power of the statistical analysis. Thus, this study should be replicated with larger samples. A comparative analysis of elderly patients with severe disease and younger patients should be performed to confirm our results and measure the impact of the severity of the illness and age, respectively, on the attitudes towards euthanasia of those age groups.

Some important considerations can be retrieved from our analysis. First, even mild depressive and anxiety symptoms negatively impact patients’ well-being, being related to loneliness feelings, worse cognitive performance, personal distress, and poorer health status. Second, the factors that most influenced a favourable attitude towards euthanasia in patients were related to personality traits, cognitive abilities, and empathic capacity (higher conscientiousness, empathic concern, and fantasy) and lower personal distress (Table 5) rather than depressive and anxiety symptoms (suggesting that, at least for this severity level, the disease does not determine a specific attitude). Finally, the low educational level, together with depressive and anxiety symptoms, might harm patients’ clairvoyance, determine poorer literacy, make access to information and health services harder, and interfere with their capacity to make well-informed and free decisions, favouring a paternalistic approach.

## 6. Conclusions

The challenge when addressing euthanasia in older adults with mixed anxiety-depressive disorder is not only whether they are fully self-determinate to request euthanasia but also if a timely and proper diagnosis, treatment, and support were at their disposal and implemented. Enhancing these patients’ sense of satisfaction and usefulness in a production-oriented society that tends to devalue and alienate them is essential. Suppose non-fatal disabilities whose unbearable suffering is either questionable and/or derived from the absence of proper psychiatric, psychosocial, and economic support become a generally accepted criterion for granting euthanasia. In that case, human rights can be put at stake.

## Figures and Tables

**Table 1:** Characterisation of the sample of patients (*n* = 114) and controls (*n* = 25).

	Patients (*n* = 114)	Controls (*n* = 25)	*p*-Value
Gender, *n* (%)			**0.001^X^**
Female	87 (76.3)	11 (44)	
Male	27 (23.7)	14 (56)	
Age (years), med [1stQ; 3rdQ], min-max	72 [68.8; 76.3], 65–91	71 [69.5; 75], 66–78	0.520^M^
Years of school, med [1stQ; 3rdQ], min-max	4 [4; 4], 0–17	6 [4; 13.5], 3–17	**<0.001^M^**
Civil status, *n* (%)			0.214^F^
Married	69 (60.5)	21 (84)	
Divorced	12 (10.5)	1 (4)	
Single	7 (6.1)	0 (0)	
Widow	26 (22.8)	3 (12)	
Religion, *n* (%)			0.112^F^
Catholic	111 (97.4)	23 (92)	
Jehovah witness	2 (1.8)	0 (0)	
Agnostic	1 (0.9)	2 (8)	
Profession, *n* (%)			1^F^
Self-employed	1 (0.9)	0 (0)	
Manager	1 (0.9)	0 (0)	
Retired	112 (98.2)	25 (100)	
Household, med [1stQ; 3rdQ], min-max	2 [2; 3], 1–11	2 [2; 2], 1–5	0.912^M^

Abbreviations: med: median; 1stQ and 3rdQ: first and third quartile; *p*-value^M^: Mann–Whitney test’s *p*-value; *p*-value^X^: Chi-square test’s *p*-value; *p*-value^F^: Fisher–Freeman–Halton test’s *p*-value; bold: statistically significant *p*-values (*p* < 0.05).

**Table 2:** Scales descriptive of patients (*n* = 114) and controls (*n* = 25).

	Baseline			Six Months Later		
Scales	Patients (*n* = 114)	Controls (*n* = 25)	*Mann–Whitney’s* *p-Value with Bonferroni Correction for Multiple Testing (33 Tests)* *	Patients (*n* = 90)	Controls (*n* = 17)	*Mann–Whitney’s* *p-Value with Bonferroni Correction for Multiple Testing (33 Tests)* *
	Med (IIQ), Min–Max	Med (IIQ), Min–Max		Med (IIQ), Min–Max	Med (IIQ), Min–Max	
HADS Depression (8–10: mild symptoms)	9 (6; 10), 0–15	1 (0; 2), 0–5	**<0.001**	8 (3; 9), 0–14	1 (0.5; 2), 0–3	**<0.001**
HADS Anxiety (8–10: mild symptoms)	8 (5; 10), 1–19	1 (0.5; 2), 0–4	**<0.001**	7 (3; 9), 2–14	1 (0; 2), 0–2	**<0.001**
MMS	28 (26; 29), 11–30	30 (29; 30), 25–30	**<0.001**	28.5 (27; 30), 15–30	30 (30; 30, 28–30	**0.001**
CDT	9 (7; 9.5), 1–10	10 (9.5; 10), 5–10	**<0.001**	9.25 (7.9; 9.5), 1–10	10 (9.5; 10), 9–10	**0.002**
UCLALs	36.5 (20; 46), 16–64	22 (17; 27.5), 16–31	**0.001**	33 (20; 41,3), 16–60	19 (16.5; 22.5), 16–25	**0.007**
MAT	5.86 (5.25; 6), 3.71–6	6 (5.4; 6), 5–6	1	5.86 (5.43; 6), 3.71–6	5.86 (5.57; 6), 5.29–6	1
BI	100 (100; 100), 70–100	100 (100; 100), 100–100	1	100 (100; 100), 75–100	100 (100; 100), 100–100	1
YATEs	83.5 (51.5; 95.25), 3–104	52 (30; 80.5), 2–103	0.288	86 (41; 97.5), 3–104	55 (23.5; 80.5), 0–103	0.590
WATEs (patient’s decision)	4 (2.75; 4.75), 1–5	3.25 (2.25; 4), 1–5	1	4 (2.4; 4.5), 1–5	4 (2; 4), 1–5	1
WATEs (doctor’s decision)	2.75 (1.5; 3.875), 1–5	2 (1.5; 2.5), 1–4	0.612	2.33 (1.7; 3.6), 1–5	2 (2; 2), 1–3	1
MDMQ						
Self-esteem (max = 12)	8 (5; 10), 1–12	10 (8; 11), 6–12	**<0.001**	8 (5.8; 10), 1–12	11 (8; 11.5), 6–12	0.315
Vigilance (max = 12)	10 (6; 12), 0–12	12 (10; 12), 2–12	1	11 (8; 12), 0–12	12 (10; 12), 3–12	1
Buck Passing (max = 12)	6 (2; 11), 0–12	3 (1.5; 6), 0–12	0.514	5 (2; 10), 0–12	4 (1; 7), 0–9	1
Procrastination (max = 10)	3 (1; 6.25), 0–10	3 (1; 4), 0–9	1	3 (1; 6), 0–10	3 (1; 4.5), 0–9	1
Hypervigilance (max = 10)	6 (3.75; 8), 0–10	4 (2; 5), 0–10	0.144	5.5 (3; 8), 0–10	4 (3; 5.5), 0–7	1
NEO-FFI						
Neuroticism	31 (20; 36.25); 2–47	16 (12; 19.5), 4–28	**<0.001**	28.5 (18; 34), 4–41	14 (13; 20), 11–25	**<0.001**
Extraversion	19.5 (16; 25.25), 8–44	31 (26; 33), 17–36	**<0.001**	20.5 (17; 26), 9–39	29 (26.5; 33), 20–36	**<0.001**
Openness	12 (7; 19), 3–39	23 (18.5; 27.5), 6–33	**<0.001**	14 (10; 19), 3–35	23 (18.5; 26.5), 14–35	**<0.001**
Agreeableness	33 (30; 35), 18–41	33 (29.5; 36.5), 20–41	1	32.5 (30; 34), 19–38	33 (30; 34.5), 24–36	1
Conscientiousness	33.5 (31; 36), 4–46	36 (34; 39.5), 31–45	**0.016**	34 (31; 36), 12–39	36 (35; 37.5), 28–41	0.064
IRI						
Perspective Taking	3.17 (2; 3.67), 0.5–4	2.5 (1.5; 3.3), 1–4	1	3.17 (1.7; 3.8), 0–4	1.67 (1.17; 3.34), 1–3.83	1
Empathic Concern	3.33 (2.63; 3.83), 1.3–4	3.2 (2.4; 3.6), 1.5–4	1	3.42 (2.8; 3.8), 1.7–4	2.83 (2.50; 3.25), 1.67–3.83	1
Personal Distress	2.83 (1.83; 3.33), 0–4.2	1.7 (0.8; 2.3), 0–3	**<0.001**	2.75 (2; 3.3), 0.5–4	1.5 (1; 2.09), 0.33–2.67	**<0.001**
Fantasy	1.5 (0.67; 2.33), 0–3.7	1.7 (1.2; 2.3), 0.3–3.3	1	1.5 (0.5; 2.2), 0–3.7	1.5 (1.09; 1.67), 0.50–3.33	1
SF36v2						
physical functioning	90 (65; 95), 10–100	95 (92.5; 100), 60–100	**<0.001**	90 (70; 90), 10–95	95 (90; 95), 50–100	**0.005**
physical role	93.75 (62.5; 100), 12.5–100	100 (100; 100), 75–100	**0.012**	100 (75; 100), 6.3–100	100 (100; 100), 100–100	**0.026**
pain	74 (62; 84), 0–100	84 (62; 100), 51–100	1	84 (62; 100), 12–100	84 (74; 100), 62–100	1
general health	38.5 (30; 50), 15–95	67 (51; 73.5), 35–100	**<0.001**	37.5 (28.8; 52), 10–87	67 (56; 74,5), 30–95	**<0.001**
vitality	37.5 (25; 62.5), 6.25–93.75	75 (71.9; 81.3), 56.25–93.75	**<0.001**	43.75 (31.3; 56.3), 0–87.5	75 (69; 81), 69–100	**<0.001**
social function	50 (25; 87.5), 0–100	100 (81.3; 100), 62.5–100	**<0.001**	62.5 (25; 90,6), 0–100	100 (100; 100), 75–100	**<0.001**
emotional role	75 (50; 100), 0–100	100 (100; 100), 83.33–100	**<0.001**	91.67 (58.3; 100), 25–100	100 (100; 100), 100–100	**0.003**
mental health	50 (35; 70), 5–100	90 (80; 90), 65–100	**<0.001**	67.5 (50; 85), 15–95	90 (85; 90), 55–100	**0.003**
TASR	7 (4; 10), 2–18	3 (2; 3), 2–8	**<0.001**	5.5 (3; 7), 3–16	3 (2; 3), 2–3	**<0.001**

Abbreviations: min—minimum; max—maximum; med—median; IIQ—interquartile range [1°Q;3°Q]; * Mann–Whitney test bilateral *p*-value; bold: statistically significant *p*-values (*p* < 0.05). HADS: hospital anxiety and depression scale; MMS: mini-mental state; CDT: clock-drawing test; UCLALs: UCLA loneliness scale; MAT: Measure Treatment Adherence; BI: Barthel index; YATEs: Yara attitude towards euthanasia scale; WATEs: Wasserman attitude towards euthanasia scale; MDMQ: Melbourne decision-making questionnaire; NEO-FFI: NEO five-factor inventory; SF36v2: short-form health survey—version 2; IRI: interpersonal reactivity index; TASR: tool for assessment of suicide risk.

**Table 3:** Descriptives and scales comparison of patients between the baseline (*n* = 114) and six months later (*n* = 90).

Scales	Baseline, Med (IIQ), Min–Max	Six Months Later, Med (IIQ), Min–Max	*Wilcoxon’s* *p-Value with Bonferroni Correction for Multiple Testing (14 Tests)*
HADS Depression	9 (6; 10), 0–15	8 (3; 9), 0–14	**<0.001**
HADS Anxiety	8 (5.8; 10), 1–19	7 (3; 9), 2–14	**<0.001**
MMS	28 (26; 29), 11–30	28.5 (27; 30), 15–30	**<0.001**
CDT	9 (6.9; 9,5), 1–10	9.25 (7.9; 9.5), 1–10	1
UCLALs	36 (20; 45), 16–62	33 (20; 41.3), 16–60	**<0.001**
MAT	5.86 (5.25; 6), 3.71–6	5.86 (5.43; 6), 3.71–6	**1**
SF36v2 Physical functioning	90 (65; 95), 10–100	90 (70; 90), 10–95	1
SF36v2 Physical role	100 (67.2; 100), 12.5–100	100 (75; 100), 6.3–100	**0.010**
SF36v2 Pain	74 (62; 91), 0–100	84 (62; 100), 12–100	0.238
SF36v2 General health	38.5 (25; 50.5), 15–87	37.5 (28,8; 52), 10–87	1
SF36v2 Vitality	37.5 (25; 62.5), 6.25–93.75	43.75 (31.3; 56.3), 0–87.5	1
SF36v2 Social function	56.2 (25; 87.5), 0–100	62.5 (25; 90.6), 0–100	**0.007**
SF36v2 Emotional role	75 (50; 100), 0–100	91.67 (58.3; 100), 25–100	**0.001**
SF36v2 Mental health	50 (35; 70), 5–100	67.5 (50; 85), 15–95	**<0.001**

Abbreviations: med—median; IIQ—interquartile range [1°Q;3°Q]; min—minimum; max—maximum; bold: statistically significant *p*-values (*p* < 0.05). HADS: hospital anxiety and depression scale; MMS: mini-mental state; CDT: clock-drawing test; UCLALs: UCLA loneliness scale; SF36v2: short-form health survey—version 2.

**Table 4:** Spearman correlations of yates with UCLALs, HADS, IRI, NEO-FFI, MDMQ, sf36-v2, and TASR in both patients’ (*n* = 114) and controls’ (*n* = 25) samples.

	Correlation with YATEs	
Scales and Subscales	Patients	Controls
UCLALs	−0.181	0.159
HADS Depression	−0.100	0.280
HADS Anxiety	−0.084	0.297
IRI Perspective taking	0.758 **	0.803 **
IRI Empathic concern	0.325 **	0.668 **
IRI Personal distress	−0.200 *	−0.128
IRI Fantasy	0.378 **	0.368
NEO-FFI Neuroticism	−0.167	0.021
NEO-FFI Extroversion	0.101	0.011
NEO-FFI Openness	0.232 *	0.012
NEO-FFI Agreeableness	0.223 *	0.188
NEO-FFI Conscientiousness	0.293 **	−0.114
MDMQ Self-esteem	0.302 **	0.260
MDMQ Vigilance	0.276 **	0.179
MDMQ Buck passing	−0.280 **	−0.384
MDMQ Procrastination	−0.371 **	−0.415 *
MDMQ Hypervigilance	−0.344 **	−0.375
SF36v2 Physical functioning	0.163	−0.227
SF36v2 Physical role	0.086	0.180
SF36v2 Pain	0.007	−0.329
SF36v2 General health	−0.060	−0.177
SF36v2 Vitality	0.116	−0.132
SF36v2 Social function	0.128	−0.050
SF36v2 Emotional role	0.089	0.172
SF36v2 Mental health	0.087	−0.149
TASR	−0.124	−0.146

Abbreviations: bold: statistically significant *p*-values (* *p* < 0.05; ** *p* < 0.01); UCLALs: UCLA loneliness scale; HADS: hospital anxiety and depression scale; MDMQ: Melbourne decision-making questionnaire; NEO-FFI: NEO five-factor inventory; SF36v2: short-form health survey—version 2; IRI: interpersonal reactivity index; TASR: tool for assessment of suicide risk.

**Table 5:** Non-standardised regression coefficients (with confidence interval and *p* value) of multiple linear regression with yates as the dependent variable for the patients’ sample (*n* = 114).

Independent Variables	Simple Linear Regression Model		Initial Multiple Model R^2^ = 0.362 F(16,97) = 3.44, *p* < 0.001		Final Multiple Model R^2^ = 0.325 F(4109) = 13.1, *p* < 0.001	
	B [I.C. a 95%]	*p*-Value	B [I.C. a 95%]	*p*-Value	B [I.C. a 95%]	*p*-Value
Age	−1.21 [−2.34; −0.08]	**0.035**	−0.33 [−1.56; 0.90]	0.598		
Gender						
*Female*	*Reference*					
*Male*	7.32 [−6.93; 21.6]	0.311				
Schooling (in years)	1.62 [−0.17; 3.42]	0.076	0.13 [−2.03; 2.29]	0.904		
HADS Depression	−0.54 [−3.45; 0.38]	0.114	−1.19 [−3.93; 1.55]	0.390		
HADS Anxiety	−0.83 [−2.47; 0.81]	0.318				
MDMQ Self-esteem	3.98 [1.97; 6.00]	**<0.001**				
MDMQ Vigilance	2.47 [0.80; 4.13]	**0.004**	0.57 [−1.59; 2.73]	0.601		
MDMQ Buck passing	−2.14 [−3.48; −0.80]	**0.002**	−0.18 [−2.13; 1.77]	0.857		
MDMQ Procrastination	−3.90 [−5.69; −2.10]	**<0.001**				
MDMQ Hypervigilance	−4.22 [−6.32; −2.13]	**<0.001**				
NEO-FFI Neuroticism	−0.54 [−1.14; 0.06]	**0.079**	0.29 [−0.87; 1.46]	0.618		
NEO-FFI Extraversion	0.48 [−0.39; 1.35]	0.276				
NEO-FFI Openness	0.90 [0.14; 1.66]	**0.020**	0.15 [−0.82; 1.12]	0.762		
NEO-FFI Agreeableness	1.60 [0.20; 2.99]	**0.025**	−0.35 [−1.87; 1.17]	0.652		
NEO-FFI Conscientiousness	2.15 [1.22; 3.09]	**<0.001**	1.65 [0.50; 2.81]	0.005	**1.65 [0.77; 2.54]**	**<0.001**
IRI Perspective taking	25.92 [22.3; 29.51]	**<0.001**				
IRI Empathic concern	15.7 [7.26; 24.2]	**<0.001**	15.79 [6.46; 25.13]	0.001	**14.32 [6.19; 22.46]**	**<0.001**
IRI Personal distress	−6.89 [−13.1; −0.67]	**0.030**	−3.21 [−11.16; 4.74]	0.425	**−6.01 [−11.78; −0.25]**	**0.041**
IRI Fantasy	11.3 [5.62; 16.9]	**<0.001**	5.54 [−1.41; 12.48]	0.117	**6.51 [1.15; 11.86]**	**0.018**
UCLALs	−0.37 [−0.80; 0.06]	**0.089**	−0.47 [−1.09; 0.16]	0.145		
SF36v2 Physical functioning	0.25 [−0.02; 0.52]	**0.069**	0.11 [−0.41; 0.57]	0.748		
SF36v2 Physical role	0.10 [−0.13; 0.33]	0.406				
SF36v2 Pain	0.09 [−0.19; 0.38]	0.515				
SF36v2 General health	−0.08 [−0.44; 0.28]	0.645				
SF36v2 Vitality	0.20 [−0.08; 0.47]	**0.159**	0.08 [−0.41; 0.57]	0.748		
SF36v2 Social function	0.15 [−0.04; 0.34]	**0.122**	−0.25 [−0.57; 0.08]	0.133		
SF36v2 Emotional role	0.13 [−0.09; 0.36]	0.234				
SF36v2 Mental health	0.11 [−0.12; 0.38]	0.296				

Abbreviations: bold: statistically significant *p*-values (*p* < 0.05); HADS: hospital anxiety and depression scale; UCLALs: UCLA loneliness scale; MDMQ: Melbourne decision-making questionnaire; NEO-FFI: NEO five-factor inventory; SF36v2: short-form health survey—version 2; IRI: interpersonal reactivity index.

## Data Availability

Data are contained within the article. Further inquiries can be directed to the corresponding author.
